# Prediction and the influencing factor study of colorectal cancer hospitalization costs in China based on machine learning-random forest and support vector regression: a retrospective study

**DOI:** 10.3389/fpubh.2024.1211220

**Published:** 2024-02-08

**Authors:** Jun Gao, Yan Liu

**Affiliations:** ^1^Department of Medical Record Statistics, Peking University Shenzhen Hospital, Shenzhen, China; ^2^School of Public Healthy, Guilin Medical University, Guilin, China

**Keywords:** colorectal cancer, hospitalization costs, influencing factors, random forest, support vector regression

## Abstract

**Aims:**

As people’s standard of living improves, the incidence of colorectal cancer is increasing, and colorectal cancer hospitalization costs are relatively high. Therefore, predicting the cost of hospitalization for colorectal cancer patients can provide guidance for controlling healthcare costs and for the development of related policies.

**Methods:**

This study used the first page of medical record data on colorectal cancer inpatient cases of a tertiary first-class hospital in Shenzhen from 2018 to 2022. The impacting factors of hospitalization costs for colorectal cancer were analyzed. Random forest and support vector regression models were used to establish predictive models of the cost of hospitalization for colorectal cancer patients and to compare and evaluate.

**Results:**

In colorectal cancer inpatients, major procedures, length of stay, level of procedure, Charlson comorbidity index, age, and medical payment method were the important influencing factors. In terms of the test set, the R2 of the Random forest model was 0.833, the R2 of the Support vector regression model was 0.824; the root mean square error (RMSE) of the Random forest model was 0.029, and the RMSE of the Support vector regression model was 0.032. In the Random Forest model, the weight of the major procedure was the highest (0.286).

**Conclusion:**

Major procedures and length of stay have the greatest impacts on hospital costs for colorectal cancer patients. The random forest model is a better method to predict the hospitalization costs for colorectal cancer patients than the support vector regression.

## 1 Introduction

Colorectal cancer(CRC) is one of the most common malignant tumors of the gastrointestinal tract, accounting for approximately 10% of all cancer deaths worldwide each year (1). Global colorectal cancer incidence is expected to increase to 25 million new cases per year by 2035 (2). CRC has become a major public health problem around the world. In China, the prevalence of colorectal cancer continues to rise each year and most patients are middle to the late stage by the time they are diagnosed (3). And the total medical expenses and the total hospitalization costs of colorectal cancer patients in China ranked second among the expenses of malignant tumors in China, second only to lung cancer (4). The surgical removal of the lesions is still an important tool in the treatment of colorectal cancer. And there are comparatively few studies related to the cost of hospitalization for operative patients. Therefore, precise cost prediction models can provide a reference for the control of hospitalization costs of colorectal cancer. According to the American Cancer Society 2020, CRC is the third most prevalent of all cancers and the second-leading cause of cancer death in the US (5). As the incidence of CRC rises, the cost of treatment overall increases accordingly. Thus, the rise in the number of people with CRC has dramatically increased the pressure on national healthcare budgets (6). The cost of treatment for CRC is not only a financial strain on patients, but also a heavy financial burden on society (7). Therefore, It is significant to control the increase of CRC hospitalization costs reasonably and effectively to reduce the financial pressure on CRC patients and the economic burden of the disease on society.

With the development of computer software and computational power, artificial intelligence has been developed in leaps forward and provides a new direction for medical diagnosis, hospital management, medical data analysis, etc. (8). Data mining is the in-depth analysis of big data to reveal significant new relationships, trends and changes. The field incorporates theories and methods from a number of subjects, including machine learning, big data, and statistics (9). Data mining is an emerging field in data research with significant value. Data mining can extract hidden information and knowledge by utilizing a variety of decisions. This is very useful for the judgment process (10). And compared to traditional statistical methods, data mining methods have fewer constraints and fewer requirements on the form of data. Therefore, data mining provides a new, effective method for accurate prediction and rational control of hospitalization costs (11). Machine learning is a division of artificial intelligence that also is an algorithm for data mining, different machine learning algorithms have different advantages (12). More and more academics are beginning to adapt machine learning algorithms to the study of hospitalization costs. Zhang and Sun (13) used a Neural Network and Support Vector Machine to predict the medical costs of breast malignant tumors. Another study (14) used Random Forest and Least Absolute Shrinkage and Selection Operator (LASSO) Regression to predict medical expenditure. However, studies using machine learning to predict the cost of hospitalization for CRC patients have not been reported to the author’s knowledge.

In recent years, China has gradually launched medical insurance payment methods for disease diagnosis-related groups (DRGs) and diagnostic intervention packages (DIPs) (15). Both DRGs and DIPs have the effect of controlling the cost of medicine and preventing excessive medical growth (16). These two payment are mainly applicable to acute hospitalized cases and are not suitable for patients with CRC. Therefore the development of precise cost prediction models can be used as a guide for reimbursement criteria for the hospitalization of patients with CRC, and can also provide a reference for reimbursement and prediction for other chronic diseases. Machine learning algorithms on hospitalization costs can also the control cost growth and prevent over-medication; it can also further detail disease groupings and explore more appropriate hospitalization cost policies for China. Thus, this research selected CRC patients from a tertiary hospital in Shenzhen as the research subject. Our study aims to apply Random Forest and Support Vector Regression to predict the cost of hospitalization and assess associated factors. Our research can provide a reference for controlling the growth of hospitalization costs and the breakdown of disease groupings.

## 2 Materials and methods

### 2.1 Data source

This study retrospectively collected data from the first page of electronic medical records of CRC patients from January 2018 to December 2022 at a tertiary hospital in Shenzhen. Criteria for inclusion: According to the International Classification of Diseases ICD-10, The study included data from the first page of cases with primary diagnosis codes C18–C20. The name of the relevant procedure is based on the name on the first page of the case. Exclusion criteria: To ensure the reliability of results, Exclude names of procedures with a number less than 20 in the main surgical operation variable. Cases with missing or repeated data on the first page of the case, or with obvious errors, are excluded, 1,590 cases were eventually included. After searching for relevant reports (17–19), We included as input variables gender, age, level of procedure, number of hospitalizations, length of stay, occupation, marital status, major procedure, Charlson Comorbidity Index(CCI) score, and the output variable was hospitalization costs (Table 1). Identification of the corresponding Charlson comorbidity by ICD-10 codes in the other diagnoses on the first page of the case and direct calculation of the CCI score (20).

**Table 1:** Variables of research.

Variables	Variable assignment	Type
Length of stay	–	Continuous variables
Gender	1 = male, 2 = female	Nominal variables
Major procedure*	1–15	Nominal variables
Marital status	1 = married, 2 = unmarried	Nominal variables
Medical payment method	1 = Basic Medical Insurance for Urban Residents, 2 = Basic Medical Insurance for Urban Workers, 3 = Self-paid，4 = Others	Nominal variables
Readmission within 1 year	1 = no, 2 = yes	Nominal variables
Admission route	1 = Emergency, 2 = Outpatient	Nominal variables
Level of procedure	1 = Level 1, 2 = Level 2, 3 = Level 3, 4 = Level 4, 5 = Others	Nominal variables
Age	–	Continuous variables
Hospitalization frequency	–	Continuous variables
Charlson comorbidity index	–	Nominal variables
Hospitalized expense	–	Continuous variables

*1, No procedure; 2, Laparoscopic partial sigmoidectomy; 3, Laparoscopic anterior rectal resection; 4, Laparoscopic sigmoid colectomy; 5, Laparoscopic right hemicolectomy; 6, Laparoscopic left hemicolectomy; 7, Colon biopsy; 8, Intravenous port implantation; 9, endoscopic rectal mucosal dissection; 10, Partial sigmoid colectomy; 11, Sigmoidcolectomy; 12, Sigmoidectomy with colonic anastomosis; 13, Laparoscopic sigmoidectomy with colonic anastomosis; 14, Radical right hemicolectomy; 15, rectal biopsy.

### 2.2 Statistical analysis

Hospitalization costs, length of stay, age, and hospitalization frequency are continuous variables. The Shapiro-Wilk test for the continuous variables in the sample data of this paper found that none of the variables follow the normal distribution. Associated studies (21) have concluded that hospital costs have special characteristics, such as the presence of large numbers of zero-cost observations, and that the distribution exhibits left-skewed and thick-tailed characteristics, and therefore it does not obey the normal distribution and variance chi-squareness. Therefore, These variables were expressed as a component ratio and median (the lower four quantiles, the upper four quantiles). Categorical variables are similarly expressed. Nonparametric testing is defined as using the sample distribution pattern to make inferences about the overall distribution pattern. Nonparametric tests are applicable when the sample data do not satisfy a normal distribution and the overall variance is unknown. Therefore, The Nonparametric testing, The Mann–Whitney U test or Kruskal-Wallis H test was applied to compare the hospitalization costs of each group. The test level *α* = 0.05.

### 2.3 Support vector regression

Traditional linear regression methods are widely used to study the influence of various factors. However, the model required the data to meet normality, variance equality, linearity, and independence. Most reports using linear regression to predict hospitalization costs do not specify the entire set of conditions to be met (13). Due to the skewed distribution of hospitalization costs for colorectal cancer patients, traditional linear regression models have limitations in the study of factors influencing hospitalization costs. The studies have shown that (14, 22) RF algorithm and SVR algorithm are more suitable for the prediction of hospital costs as compared to other algorithms. And no study has compared RF algorithms to SVR algorithms in the prediction of the cost of hospitalization. Therefore, the RF algorithm and SVR algorithm were selected in this study for modeling and comparative. Some research shows that machine learning approaches are more applicable to the study of big data in healthcare, and other studies have shown that SVR models have a strong generalization ability in hospitalization cost prediction compared to other machine learning algorithms (22). Therefore, this study introduces support vector regression(SVR) models to explore the factors influencing hospitalization costs. SVR is a supervised machine learning model for regression. SVR has good regression performance for non-linear, high-dimensional problems (23). The main thought behind SVR is to map the data to higher dimensions and to perform regression predictions in higher dimensions. In terms of SVR, the Radial basis kernel function (RBF) is a great selection, RBF is a non-linear projection that deals well with the problem of non-linear data (24). The optimal SVR model can then be constructed by adjusting the parameters after the RBF has been selected.

### 2.4 Random forest

Random forest is also a suitable model appropriate for our study (25). A study has shown that RF has better accuracy, sensitivity and specificity compared to other machine learning algorithms such as decision trees (26). Random forest is a supervised machine learning model and has the ability to be constraint-free, requiring only adjustments to a few parameters to reach accurate predictions and the advantage of handling a wide range of quantitative and qualitative data (27). Random forest regression models perform random return sampling of samples and sample characteristics, generating a number of least squares regression trees, the most desirable results are output by referencing all least squares regression trees (27).

### 2.5 Prediction performance evaluation

For the regression problem, the values of the root mean square error RMSE, the mean absolute percentage error MAPE, or the mean absolute error MAE are frequently used to evaluate the predictive performance of the model, and the coefficient of determination R2, is used to evaluate the fit of the model. The R2 is a statistical index used to measure how well a regression model fits the observed data. The standard for the r2 value is generally set at 0.75, and the R2 value greater than 0.75 indicates that the model is well-fitted. The MAE represents the mean of the absolute value of the error between the predicted value and the actual value. The MAPE is used to reflect the extent of the data discretization. The RMSE indicates the error between the actual value and the predicted value, and it also indicates the degree of discretization between the two errors. The closer the three indicators are to 0, the better they are. The three values are the deviation of the sample value from the predicted value, which is affected by the size of the sample value and the size of the predicted value. So there is no established standard. Because the RMSE has a wider scope of evaluation, the RMSE was selected as the one of the assessment indicators in this study. In summary, the coefficient of determination R2 and the values of the root mean square error RMSE were selected as evaluation indicators in this study.

### 2.6 Software realization

Non-parametric tests are performed using IBM SPSS Statistics 25. RF models and SVR models were implemented using a package such as “e1071,” “caret,” “random forest,” etc. in R-4.0.2 software.

## 3 Results

### 3.1 Basic information

The study included 1,590 patients with CRC; Males and females made up 60.5 and 39.5% of the sample, respectively. Inpatients with a length of stay of 11–15 days made up 36% of the study population and patients with a length of stay of 20 days or longer represented only 16.7% of the study population. The main operative operations can be divided into laparoscopic treatment and non-laparoscopic treatment, with laparoscopic right hemicolectomy being the most common operation, accounting for 16.5% of the main operations with 262 cases. Unmarried people made up 11.9% of the sample, and married people 88.1% of the sample. The proportion of urban employees’ basic medical insurance made up the highest at 27.9% and the proportion of urban residents’ basic medical insurance made up the smallest at 15.3%. The number of patients readmitted within 1 year was 124 (7.8%). The majority of patients’ cases were outpatients (95.0%) with 1,461 patients. Among the surgical levels, the largest proportion of surgeries was grade 4 at 68% and the smallest was grade 1 at 2.6%. In terms of age of inpatients, 51.3% were aged 30–65 years, 37.8% were over 66 years and 10.9% were aged 19–35 years. 70.4% of patients were first-time admissions. Patients had a maximum of 31.0% of Charlson comorbidity scores in the 0–2 range and a minimum of 21.2% in the larger than or equal to 9 range. Results are shown in Table 2.

**Table 2:** Basic characteristics and univariate analysis in hospitalization costs of colorectal cancer.

Variables	No. of cases	Component ratio	Hospitalization costs [M(p25,p75)]	Test statistic	*P*
		%		(U/H)	
Gender				0.881	0.375
Male	964	60.5	66,830(28,275,79,776)		
Female	626	39.5	67,645(51,443,78,328)		
Length of stay				742.380	<0.01
1–10	346	21.7	9,262(6,271,22,344)		
11–15	573	36	65,951(57,416,73,372)		
16–20	407	25.6	73,174(65,611,82,151)		
≥21	264	16.7	86,851(74,489,82,150)		
Major procedure				944.167	<0.01
No procedure	160	10.1	7,326(4,469,12,735)		
Laparoscopic partial sigmoidectomy	147	9.2	69,473(61,140,77,981)		
Laparoscopic anterior rectal resection	177	11.1	73,124(67,232,84,257)		
Laparoscopic sigmoid colectomy	84	5.2	70,098(63,572,78,651)		
Laparoscopic right hemicolectomy	262	16.5	76,610(67,865,88,044)		
Laparoscopic left hemicolectomy	70	4.4	72,667(66,190,86,032)		
Colon biopsy	77	4.8	11,682(7,021,20,519)		
Intravenous port implantation	79	5.0	23,042(15,626,27,735)		
Endoscopic rectal mucosal dissection	21	1.3	18,692(14,735,23,138)		
Partial sigmoid colectomy	32	2.0	79,330(63,418,97,498)		
Sigmoid colectomy	44	2.8	72,445(59,756,103,772)		
Sigmoidectomy with colonic anastomosis	51	3.2	72,100(65,164,82,951)		
Laparoscopic Sigmoidectomy with colonic anastomosis	221	13.9	71,232(65,717,82,763)		
Radical right hemicolectomy	69	4.3	67,422(57,419,73,759)		
Rectal biopsy	96	6.0	10,393(7,051,20,998)		
Marital status				1.340	0.18
Married	1,400	88.1	67,356(39,695,78,829)		
Unmarried	190	11.9	66,042(17,479,78,970)		
Medical payment method				27.025	<0.01
Basic Medical Insurance for Urban Residents	243	15.3	68,567(49,601,78,805)		
Basic Medical Insurance for Urban Workers	443	27.9	65,179(28,496,76,720)		
Self-paid	285	17.9	63,399(19,664,76,972)		
Others	619	38.9	69,820(54,034,81,051)		
Readmission within 1 year				0.304	0.762
Yes	124	7.8	68,638(38,299,82,714)		
No	1,466	92.2	67,221(32,369,78,678)		
Admission route				1.159	0.247
Emergency	125	7.9	72,090(41,314,119,360)		
Outpatient	1,461	92.1	68,213(46,722,82,058)		
Level of procedure				801.268	<0.01
1	42	2.6	24,466(17,045,29,079)		
2	233	14.7	17,359(8,293,28,060)		
3	74	4.7	66,856(56,280,75,749)		
4	1,081	68.0	72,597(56,280,84,383)		
Others	160	10.0	7,326(4,469,12,735)		
Age				11.869	<0.01
19–35	174	10.9	64,495(24,765,71,665)		
36–65	815	51.3	66,745(49,601,77,349)		
≥66	601	37.8	69,659(27,953,82,608)		
Hospitalization frequency				5.434	0.246
1	1,119	70.4	66,516(33,717,78,087)		
2	179	11.2	69,467(28,071,81,965)		
3	57	3.6	69,659(42,658,81,947)		
4	70	4.4	71,571(59,683,80,982)		
≥5	165	10.4	70,362(20,972,85,991)		
CCI score				290.916	<0.01
0–2	493	31.0	55,668(9,742,68,281)		
3–5	376	23.6	65,999(27,939,77,806)		
6–8	385	24.2	69,098(60,552,78,250)		
≥9	336	21.2	78,389(68,782,98,061)		

### 3.2 Analysis of differences of hospitalization costs for CRC patients

The Mann–Whitney U test and Kruskal-Wallis H test with hospitalization cost as the output variable. As shown in Table 2. There were no statistical differences in hospitalization costs with gender (*p* = 0.375), marital status(*p* = 0.18), whether readmitted within 12 months (*p* = 0.762), route of admission (*p* = 0.247), or the number of hospitalizations (*p* = 0.246). The cost of hospitalization was statistically different from the number of days in the hospital, major procedure, payment method, level of procedure, age, and CCI index (*p* < 0.05).

### 3.3 Comparison within groups

Comparison of patient’s costs for factors within variables after adjusting for alpha levels using the Bonferroni method.

As shown in Table 3, among the major procedure variables, statistical difference in hospital costs between those who did not undergo the procedure and those who had the procedure of a treatment nature; the Statistically significant difference in hospital costs between patients undergoing procedures of an investigative nature and those undergoing procedures of a treatment nature; in addition to endoscopic rectal mucosal dissection, no statistically significant difference in hospital costs between procedures of a treatment nature. Procedures of an investigative nature include Colon biopsy and rectal biopsy. Procedures of an treatment nature include Laparoscopic partial sigmoidectomy, laparoscopic anterior rectal resection, laparoscopic sigmoidectomy, laparoscopic right hemicolectomy, laparoscopic left hemicolectomy, sigmoidectomy with colonic anastomosis, laparoscopic sigmoidectomy with colonic anastomosis, radical right hemicolectomy, partial sigmoidectomy. Comparison of the number of days in hospital Internal variables, all differences in hospitalization costs between all internal variables were statistically significant, this means that the longer the hospital stay, the larger the hospital costs. Comparison of internal variables of CCI score, all differences in CCI score between all internal variables were statistically significant, this means that the larger the CCI score, the larger the hospital costs. There is a statistical difference between the cost of hospitalization for the level 3 procedure and other levels of procedure. There is a statistical difference between the age of 19–35 years and Greater than or equal to 66 years of age. There is a statistically significant difference in the cost of hospitalization between patients paying out of pocket and other payment methods.

**Table 3:** Significance results of pairwise comparison.

Variables (pairwise comparisons between groups)	Test statistic (χ^2^)	*p*	Adjust *p*
Major procedure			
No procedure vs. laparoscopic partial sigmoidectomy	728.520	<0.01	<0.01
No procedure vs. laparoscopic anterior rectal resection	859.884	<0.01	<0.01
No procedure vs. laparoscopic sigmoidectomy	772.679	<0.01	<0.01
No procedure vs. laparoscopic right hemicolectomy	919.103	<0.01	<0.01
No procedure vs. laparoscopic left hemicolectomy	848.371	<0.01	<0.01
No procedure vs. partial sigmoidectomy	890.062	<0.01	<0.01
No procedure vs. sigmoidectomy	842.432	<0.01	<0.01
No procedure vs. sigmoidectomy with colonic anastomosis	829.167	<0.01	<0.01
No procedure vs. laparoscopic sigmoidectomy with colonic anastomosis	816.215	<0.01	<0.01
No procedure vs. radical right hemicolectomy	873.714	<0.01	<0.01
Colon biopsy vs. laparoscopic partial sigmoidectomy	678.994	<0.01	<0.01
Colon biopsy vs. laparoscopic anterior rectal resection	810.358	<0.01	<0.01
Colon biopsy vs. laparoscopic sigmoidectomy	723.153	<0.01	<0.01
Colon biopsy vs. laparoscopic right hemicolectomy	869.557	<0.01	<0.01
Colon biopsy vs. laparoscopic left hemicolectomy	798.845	<0.01	<0.01
Colon biopsy vs. sigmoidectomy with colonic anastomosis	805.412	<0.01	<0.01
Colon biopsy vs. laparoscopic sigmoidectomy with colonic anastomosis	792.460	<0.01	<0.01
Colon biopsy vs. radical right hemicolectomy	642.967	<0.01	<0.01
Intravenous port implantation vs. laparoscopic partial sigmoidectomy	623.415	<0.01	<0.01
Intravenous port implantation vs. Laparoscopic anterior rectal resection	713.191	<0.01	<0.01
Intravenous port implantation vs. laparoscopic sigmoidectomy	739.895	<0.01	<0.01
Intravenous port implantation vs. laparoscopic right hemicolectomy	759.831	<0.01	<0.01
Intravenous port implantation vs. laparoscopic left hemicolectomy	727.996	<0.01	<0.01
Intravenous port implantation vs. sigmoidectomy with colonic anastomosis	669.251	<0.01	<0.01
Intravenous port implantation vs. laparoscopic sigmoidectomy with colonic anastomosis	682.473	<0.01	<0.01
Intravenous port implantation vs. radical hemicolectomy	520.029	<0.01	<0.01
Intravenous port implantation vs. radical right hemicolectomy	727.021	<0.01	<0.01
Intravenous port implantation vs. sigmoidectomy	695.738	<0.01	<0.01
Intravenous port implantation vs. partial sigmoidectomy	743.369	<0.01	<0.01
Endoscopic rectal mucosal dissection vs. laparoscopic partial sigmoidectomy	656.942	<0.01	<0.01
Endoscopic rectal mucosal dissection vs. laparoscopic anterior rectal resection	746.718	<0.01	<0.01
Endoscopic rectal mucosal dissection vs. laparoscopic sigmoidectomy	770.422	<0.01	<0.01
Endoscopic rectal mucosal dissection vs. laparoscopic right hemicolectomy	793.358	<0.01	<0.01
Endoscopic rectal mucosal dissection vs. laparoscopic left hemicolectomy	761.492	<0.01	<0.01
Endoscopic rectal mucosal dissection vs. sigmoidectomy with colonic anastomosis	716.000	<0.01	<0.01
Endoscopic rectal mucosal dissection vs. laparoscopic sigmoidectomy with colonic anastomosis	703.048	<0.01	<0.01
Endoscopic rectal mucosal dissection vs. radical hemicolectomy	553.556	<0.01	<0.09
Endoscopic rectal mucosal dissection vs. radical right hemicolectomy	760.548	<0.01	<0.01
Endoscopic rectal mucosal dissection vs. sigmoidectomy	729.265	<0.01	<0.01
Endoscopic rectal mucosal dissection vs. partial sigmoidectomy	776.896	<0.01	<0.01
Rectal biopsy vs. laparoscopic partial sigmoidectomy	730.609	<0.01	<0.01
Rectal biopsy vs. laparoscopic anterior rectal resection	820.384	<0.01	<0.01
Rectal biopsy vs. laparoscopic sigmoidectomy	844.089	<0.01	<0.01
Rectal biopsy vs. laparoscopic right hemicolectomy	867.025	<0.01	<0.01
Rectal biopsy vs. laparoscopic left hemicolectomy	835.159	<0.01	<0.01
Rectal biopsy vs. sigmoidectomy with colonic anastomosis	789.669	<0.01	<0.01
Rectal biopsy vs. laparoscopic sigmoidectomy with colonic anastomosis	776.715	<0.01	<0.01
Rectal biopsy vs. radical hemicolectomy	834.214	<0.01	<0.01
Rectal biopsy vs. radical right hemicolectomy	802.932	<0.01	<0.01
Rectal biopsy vs. sigmoidectomy	764.855	<0.01	<0.01
Rectal biopsy vs. partial sigmoidectomy	850.562	<0.01	<0.01
Length of stay			
1–10 vs. 11–15	503.473	<0.01	<0.01
1–10 vs. 16–20	696.180	<0.01	<0.01
1–10 vs. ≥21	955.871	<0.01	<0.01
11–15 vs. 16–20	192.707	<0.01	<0.01
11–15 vs. ≥21	452.397	<0.01	<0.01
16–20 vs. ≥21	256.691	<0.01	<0.01
Level of procedure			
2 vs. 4	675.056	<0.01	<0.01
2 vs. 5	157.792	<0.01	<0.01
5 vs. 4	832.848	<0.01	<0.01
3 vs. 1	482.661	<0.01	<0.01
3 vs. 2	491.830	<0.01	<0.01
3 vs. 4	183.226	<0.01	<0.01
3 vs. 5	649.622	<0.01	<0.01
CCI score			
0–2 vs. 3–5	202.591	<0.01	<0.01
0–2 vs. 6–8	305.054	<0.01	<0.01
0–2 vs. ≥9	542.379	<0.01	<0.01
3–5 vs. 6–8	102.463	<0.01	0.013
3–5 vs. ≥9	340.149	<0.01	<0.01
6–8 vs. ≥9	237.685	<0.01	<0.01
Age			
1 vs. 3	134.137	<0.01	0.02
Medical payment method			
3 vs. 4	144.775	<0.01	<0.01

### 3.4 Model construction and parameter tuning

**Figure 1:**
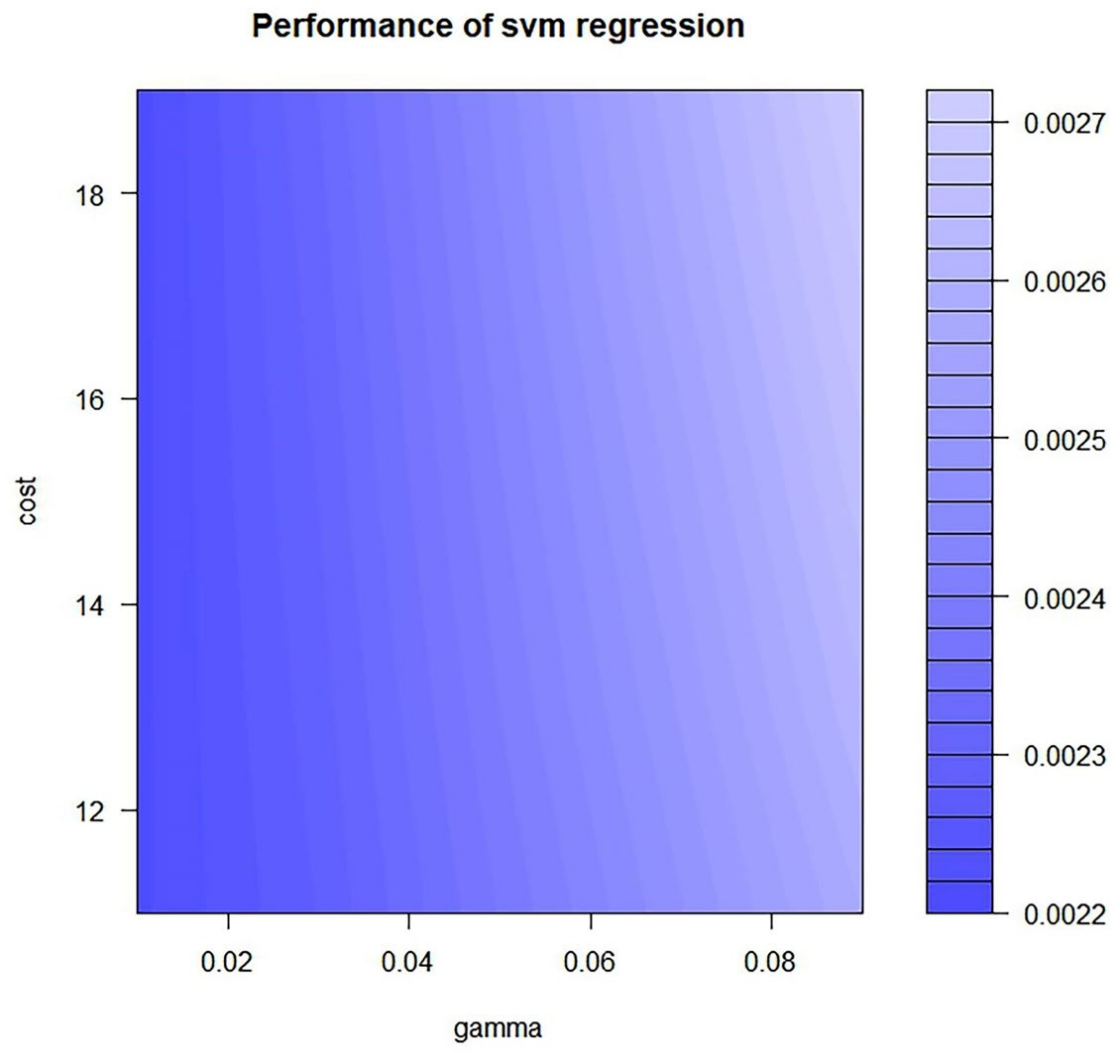
SVR model tuning.

We integrated all variables into the RF model and the SVR model. To reduce the impact of unit differences between different variables, we have normalized the variables using the linear conversion function, The formula is as follows: y = (x-Minx)/(Maxx-Minx). After we included all the variables in RF model and SVR model we found that the R2 value of the RF model is 0.65 and the R2 value of the SVR model is 0.54, it shows that both the models are poorly fitted. According to the effect of the input variables on the output variables only the top six variables were found to have greater effect on the hospitalization costs. Therefore, the model was further adjusted by reducing one variable at a time, and it was eventually found that the model had the best performance when the number of variables was 6. Finally, selected as input variables were age, length of stay, major procedure, medical payment method, CCI score, and level of procedure. Hospitalization costs as the output variable. The resulting parameters are determined as follows, The study used a grid search approach to parameter-tuning the RF and SVR models. The important parameters in the RF model are the number of variables to be sampled for each tree mtree and the number of decision trees to be constructed ntree. The grid search method can be made to achieve global optimality of parameters. Ntree range is set to 10–500, step size set to 1, ntree range is set to 10–500, step size set to 1. Build a model of 2,450, with R2 greatest when ntree = 142 and mytree = 4. The RBF kernel function is selected for the SVR model and the kernel function coefficients gamma and penalty function cost are adjusted using a grid search method. Gamma range is set to 0.01–0.1, the step size is set to 0.02, the cost range is set to 11–20, and the step size is set to 2. The model accuracy is highest when gamma = 0.01, and cost = 19, as shown in Figure 1.

### 3.5 Comparison of random forest and support vector regression

Both random forests and support vector regression were used with 70% of colorectal cancer patients as the training set and 30% of patients as the test set. In terms of training sets, the R2 of the RF model was 0.912, the R2 of the SVR model was 0.777; the RMSE of the RF model was 0.025, and the RMSE of the SVR model was 0.041, the prediction accuracy and the fitting effect of the RF prediction model were better than that of the SVR model. In terms of training sets, the R2 of the RF model was 0.833, the R2 of the SVR model was 0.824; the RMSE of the RF model was 0.029, and the RMSE of the SVR model was 0.032. In conclusion, both the RF and SVR models have the superior predictive ability in regression problems. in terms of the train set, the R2 value of the RF model is significantly higher than the R2 value of the SVR model, and the RMSE value of the RF model is lower than the RMSE value of the SVR model. in terms of the test set, The R2 values of the RF model are slightly higher than the R2 values of the SVR model, and there is little difference between the RMSE values of the RF model and the SVR model. In terms of the prediction of the hospitalization cost of colorectal cancer, As determined by the combined results of the train set and the test set, the prediction accuracy and the fitting effect of the RF prediction model were slightly better than that of the SVR model. As shown in Table 4.

**Table 4:** Comparison of prediction capacity of random forest model and support vector regression model.

	Random forest		Support vector regression	
	Train set	Test set	Train set	Test set
*R* ^2^	0.912	0.833	0.777	0.824
RMSE	0.025	0.029	0.041	0.032

### 3.6 Ranking the importance of variables

In the RF model, the Major procedure had the highest weight (0.283), followed by the length of stay (0.260), and the variable with the smallest is the mode of payment. In the SVR model, only five variables had weights. The major procedure had the highest weight (0.702), followed by the length of stay (0.148). The medical payment method had a weight of zero. As shown in Table 5.

**Table 5:** Importance ranking of variables.

Mode	The variable weight of RF	The variable weight of SVR
Major procedure	0.283	0.702
length of stay	0.260	0.148
Level of procedure	0.144	0.121
CCI score	0.073	0.015
Age	0.033	0.014
Medical payment method	0.017	<0.001

## 4 Discussion

Our findings show that major procedures, length of stay, level of procedure, CCI score, age, and medical payment method have statistically significant effects on hospital costs for inpatients with CRC. Gender, admission route, and Hospitalization frequency no statistically significant effects on hospital costs for inpatients with CRC, but the findings of Jacobs et al. (28) show a correlation between the admission route of inpatients and the costs of hospitalization. This could be due to the sample studied, the research is a single center-based hospitalization cost forecasting study, future multi-center studies can be done, and further, investigate the effect of admission route on hospital costs for inpatients with CRC. The related study shows (29) that multiple linear regression fits the nonlinear relationship across variables by including dummy variables. It is also possible to use the two-part model to improve the fit of the model. However, as the sample size increases, these methods provide limited effects. Springer et al. (30) study used multivariate regression to find that complications were the most important influencing factor. Our study showed complications to be third in the importance list. This may be related to the study methods and sample size. Significant values of predicted variables based on the RF prediction models, for patients with CRC, the main influencing factor for hospitalization expenses is the major procedure and the length of stay, which is consistent with the research results of Wu et al. (31) The study by Gao et al. (4) showed that the length of stay and the primary treatment and Medicare payment method were important factors influencing the cost of hospitalization for colorectal cancer patients, which is also consistent with our findings.

Combining the important values of the predictive variables of the RF prediction model and the SVR regression model and the results of related analyses. The results show that the major procedure has the most important impact on the cost of hospitalization for CRC patients, with the length of stay ranking second. The major procedure for colorectal cancer patients are grouped into 15 categories, there was a significant difference in hospitalization costs between rectal surgery performed endoscopically and laparoscopically, there is a significant difference in hospital costs for procedures of an investigative nature such as rectal biopsy, colon biopsy and procedures of a treatment type such as colorectal resection. The procedure is the most important treatment for colorectal cancer. Although the traditional open procedure can be effective, it can be highly damaging to the patient’s body, and not conducive to the patient’s own recovery (32). As minimally invasive techniques develop, the laparoscopic procedure is beginning to become the main procedure for the treatment of colorectal cancer. Laparoscopic procedures have the advantages of less trauma, less damage to surrounding tissues, and faster postoperative recovery. Laparoscopic surgery is not only effective in reducing the overall cost of medical care during hospitalization but also in reducing the length of stay, thus increasing the efficiency of hospital operations (33). However, disposable consumables and the cost of the procedure are more expensive for laparoscopic surgery than for open surgery (34). Comparisons within a hospital length of stay groups found statistical differences between hospital costs for all days of stay, the longer the hospital stay, the higher the cost of hospitalization. The study found that hospital costs were not significantly different between patients who had laparoscopic surgery and those who had open surgery, except for endoscopic rectal mucosal dissection. The reason for the speculation is that although laparoscopic surgery is expensive in terms of consumables, the smaller incision and shorter recovery period for patients reduces the number of hospital days, whereas open patients have a longer recovery period and therefore the difference in hospital costs between the two is not significant. Therefore, in addition to rectal cancer surgery, when performing a procedure for CRC, clinicians can choose the procedure with better treatment results. When performing a procedure for rectal cancer, the surgeon should consider not only the severity of the patient’s disease but also the patient’s financial situation to choose the best treatment option.

In our study, the CCI score is positively correlated with hospitalization costs. Comparison within the CCI group found significant differences in hospital costs at all levels of the CCI score. The greater the CCI score is, the more complications are; therefore, the more serious the patient’s disease, the higher the cost of diagnosis and treatment, and also the longer the hospital stay, thus affecting the total cost of hospitalization. Which is in agreement with the findings of Zhang’s research (35). CCI score is a notable influencing factor. In the future, the relationship between the combined benefits of comorbidities as well as major procedures and length of stay can be studied to determine a reasonable length of stay to prevent the effects of treatment from being compromised by too short a stay. While preventing excessive increases in hospital costs and unreasonable resource allocation due to excessive length of stay.

Age is an influential factor in the cost of hospitalization for people with CRC. The results show a statistical difference in hospitalization costs between patients aged 19–35 years and patients aged ≥66 years, which may be due to the fact that older people are physically weaker than younger people, recover more slowly consume healthcare resources, and stay in hospital for longer periods (36). Based on the significance results of pairwise comparison, medical payment method and level of procedure had an effect on hospitalization costs for CRC patients, but the impact was not clear, it is consistent with the results of the significant values and correlation analysis of the predictor variables of the RF prediction model and the SVR regression model.

### 4.1 Clinical implications

Doctors can rationalize the treatment of patients after understanding their various conditions, considering the patients’ financial situation. Hospitals or policymakers can use the model to predict colorectal cancer hospitalization costs and create individualized, precise hospital reimbursement plans to provide reference for value-based care.

## 5 Conclusion

The study shows that for patients with colorectal cancer, hospitalization costs are influenced by a number of variables, including major procedure, length of stay, CCI score, level of procedure, age, and medical payment method, with major procedure and length of stay being the most consequential variables. The hospitalization costs for procedures of an investigative nature are lower than hospitalization costs for procedures of a therapeutic nature. There are no significant difference in the cost of hospitalization between procedures of a treatment nature, with the exception of endoscopic rectal procedures. The reason for the speculation is that although laparoscopic surgery is expensive in terms of consumables, the smaller incision and shorter recovery period for patients reduce the number of hospital days. Whereas open patients have a longer recovery period. Therefore, the difference in hospital costs between the two is not significant. The further research is required to substantiate this. The CCI score is an important factor in hospitalization costs, As the number of comorbidities increases, the cost of hospitalization for CRC patients increases. The hospitalization cost prediction model constructed by the RF algorithm is better than the hospitalization cost prediction model constructed by the SVR algorithm. The RF model can predict hospitalization costs for CRC patients, the model can provide an effective strategy for Medicare to consider the implementation of personalized and precise hospitalization reimbursement schemes in the future.

## 6 Limitation

Our study has some limitations. Due to the limitations of the conditions, the study was conducted using the patient data from only one hospital. Future studies can further expand the sample size and the sample range and conduct more in-depth studies. This paper is the study of the hospitalization costs based on the first page of the medical recard. The dependent variables included in this study are limited. Future studies could incorporate more dependent variables.

## Data availability statement

The original contributions presented in the study are included in the article/supplementary material, further inquiries can be directed to the corresponding author.

## Ethics statement

Ethical approval was not required for the study involving humans in accordance with the local legislation and institutional requirements. Written informed consent to participate in this study was not required from the participants or the participants’ legal guardians/next of kin in accordance with the national legislation and the institutional requirements. The manuscript presents research on animals that do not require ethical approval for their study.

## Author contributions

YL and JG designed the study together. YL provided the data, funding, and conceptualization guidance. JG performed data analysis and wrote the manuscript. All authors contributed to the article and approved the submitted version.

## References

[1] Dekker E Tanis PJ Vleugels JLA Kasi PM Wallace MB. Colorectal cancer. Lancet. (2019) 394:1467–80. doi: 10.1016/s0140-6736(19)32319-0 31631858

[2] Araghi M Soerjomataram I Jenkins M Brierley J Morris E Bray F. Global trends in colorectal cancer mortality: projections to the year 2035. Int J Cancer. (2019) 144:2992–3000. doi: 10.1002/ijc.32055 30536395

[3] Cao M Li H Sun D Chen W. Cancer burden of major cancers in China: a need for sustainable actions. Cancer Commun. (2020) 40:205–10. doi: 10.1002/cac2.12025 PMC7667573 32359212

[4] Yuan G-L Liang L-Z Zhang Z-F Liang Q-L Huang Z-Y Zhang H-J. Hospitalization costs of treating colorectal cancer in China: a retrospective analysis. Medicine. (2019) 98:33. doi: 10.1097/MD.0000000000016718 PMC6831262 31415365

[5] Viale PH. The american cancer society's facts and figures: 2020 edition. J Adv Pract Oncol. (2020) 11:135–6. doi: 10.6004/jadpro.2020.11.2.1 33532112 PMC7848816

[6] Lemmon E Hanna CR Hall P Morris EJA. Health economic studies of colorectal cancer and the contribution of administrative data: a systematic review. Eur J Cancer Care. (2021) 30:e13477. doi: 10.1111/ecc.13477 34152043

[7] Qu RZ Ma YP Zhang ZP Fu W. Increasing burden of colorectal cancer in China. Lancet Gastroenterol Hepatol. (2022) 7:700. doi: 10.1016/S2468-1253(22)00156-X 35809603

[8] Sun S Tan ET Mintz DN Sahr M Endo Y Nguyen J. Evaluation of deep learning reconstructed high-resolution 3D lumbar spine MRI. Eur Radiol. (2022) 32:6167–77. doi: 10.1007/s00330-022-08708-4 35322280

[9] Yu TZ He Z Zhou QH Ma J Wei LH. Analysis of the factors influencing lung cancer hospitalization expenses using data mining. Thoracic Cancer. (2015) 6:338–45. doi: 10.1111/1759-7714.12147 26273381 PMC4448379

[10] Thuraisingham B. Data mining: Technologies, techniques, tools and trends. Boca Raton, FL: CRC Press (1999).

[11] Margolis R Derr L Dunn M Huerta M Larkin J Sheehan J. The national institutes of health’s big data to knowledge (bd2k) initiative: capitalizing on biomedical big data. J Am Med Inform Assoc. (2014) 21:957–8. doi: 10.1136/amiajnl-2014-002974 25008006 PMC4215061

[12] Pedersen CF Andersen MO Carreon LY Eiskjaer S. Applied machine learning for spine surgeons: predicting outcome for patients undergoing treatment for lumbar disc herniation using PRO data. Glob Spine J. (2022) 12:866–76. doi: 10.1177/2192568220967643 PMC9344505 33203255

[13] Zhang J Sun L. Analysis of influencing factors on hospitalization expenses of patients with breast malignant tumor undergoing surgery: based on the neural network and support vector machine. J Healthc Eng. (2021) 2021:9268660. doi: 10.1155/2021/9268660 34868533 PMC8635896

[14] Dai P Chang W Xin Z Cheng H Ouyang W Luo A. Retrospective study on the influencing factors and prediction of hospitalization expenses for chronic renal failure in China based on random forest and LASSO regression. Front Public Health. (2021) 9:678276. doi: 10.3389/fpubh.2021.678276 34211956 PMC8239170

[15] He AJ. Scaling-up through piloting: dual-track provider payment reforms in China's health system. Health Policy Plan. (2022) 38:218–27. doi: 10.1093/heapol/czac080 PMC9923375 36103333

[16] Liao ZY Liu B. A comparison of the differences between Medicare DRG and DIP payment methods. Chin Hosp Direct. (2022) 18:77–81.

[17] Sougklakos I Athanasiadis E Boukovinas I Karamouzis M Koutras A Papakotoulas P. Treatment pathways and associated costs of metastatic colorectal cancer in Greece. Cost Eff Resour AllocE. (2022) 20:7. doi: 10.1186/s12962-022-00339-2 PMC8842737 35164784

[18] Liu Q Yu Y-K Wang D-Y Xing W-Q. Factors associated with the costs of hospitalization after esophagectomy: a retrospective observational study at a three-tertiary cancer hospital in China. J Thorac Dis. (2020) 12:5970–9. doi: 10.21037/jtd-20-2770 33209429 PMC7656344

[19] Garg R Cheng V Ellis U Verma V McTaggart-Cowan H Peacock S. Direct medical costs of young-onset colorectal cancer: a worldwide systematic review. BMC Health Serv Res. (2020) 22:1100. doi: 10.1186/s12913-022-08481-6 PMC9426038 36042470

[20] Wang N Huang YQ Fei XL Wei L Chen H. An analysis of the association between Charlson comorbidity and in-hospital mortality in stroke patients based on the first page of the inpatient case. Health Q Manag China. (2018) 25:20–3. doi: 10.13912/j.cnki.chqm.2018.25.3.08

[21] Jones AM. Models for health care. HEDG working paper The University of York (2010).

[22] Wu CY Zha DH Gao H. Prediction of bronchopneumonia inpatients' total hospitalization expenses based on BP neural network and support vector machine models. Comput Math Methods Med. (2022) 2022:9275801. doi: 10.1155/2022/9275801 35633928 PMC9132643

[23] Kadyrova NO Pavlova LV. Comparative efficiency of algorithms based on support vector Machines for Regression. Biofizika. (2015) 60:1085–98. doi: 10.1134/S0006350915060111 26841501

[24] Ding S Zhang N Zhang X Wu F. Twin support vector machine: theory, algorithm and applications. Neural Comput Applic. (2017) 28:3119–30. doi: 10.1007/s00521-016-2245-4

[25] Xu Y Greene TH Bress AP Bellows BK Zhang Y Zhang Z. An efficient approach for optimizing the cost-effective individualized treatment rule using conditional random forest. Stat Methods Med Res. (2022) 31:2122–36. doi: 10.1177/09622802221115876 35912490

[26] EsmailyHTayefiMDoostiHGhayour-MobarhanMNezamiHAmirabadizadehA. Comparison between decision tree and random forest in determining the risk factors associated with type 2 diabetes. J Res Health Sci. (2018) 18:e0041229784893

[27] Rigatti SJ. Random forest. J Insur Med. (2017) 47:31–9. doi: 10.17849/insm-47-01-31-39.1 28836909

[28] Jacobs MA Tetley JC Kim J Schmidt S Brimhall BBB Mika V. Association of cumulative colorectal surgery hospital costs, readmissions, and emergency department/observation stays with insurance type. J Gastrointest Surg. (2023) 27:965–79. doi: 10.1007/s11605-022-05576-7 36690878 PMC10133377

[29] Mihaylova B Briggs A Hagan A Thompson SG. Review of statistical Methodsfor analysing healthcare resources and costs. Health Econ. (2011) 20:897–916. doi: 10.1002/hec.1653 20799344 PMC3470917

[30] Springer JE Doumouras AG Saleh F Lee J Cadeddu M Eskicioglu C. Drivers of inpatient costs after colorectal surgery within a publicly funded healthcare system. Dis Colon Rectum. (2019) 6:747–54. doi: 10.1097/DCR.0000000000001309 31094961

[31] Wu SW Pan Q Chen T. Research on diagnosis-related group grouping of inpatient medical expenditure in colorectal cancer patients based on a decision tree model. World J Clin Cases. (2020) 8:2484–93. doi: 10.12998/wjcc.v8.i12.2484 32607325 PMC7322429

[32] Gu JY Zhang XX Yang YF Guo HC Cheng W Liu J. Analysis of factors influencing hospitalization costs of colorectal cancer surgery patients based on quantile regression model. World Sci Technol. (2022) 24:4520–7.

[33] van den Brink MM Tweed TTT de Hoogt PA Hoofwijk AGM Hulsewe KWE Sosef MN. The introduction of laparoscopic colorectal surgery: can it improve hospital economics? Dig Surg. (2021) 38:58–65. doi: 10.1159/000511180 33171465

[34] Mar J Anton-Ladislao A Ibarrondo O Arrospide A Lazaro S Gonzalez N. Cost-effectiveness analysis of laparoscopic versus open surgery in colon cancer. Surg Endosc Other Interv Tech. (2018) 32:4912–22. doi: 10.1007/s00464-018-6250-9 29869084

[35] Zhang X Wang X Wang M Gu J Guo H Yang Y. Effect of comorbidity assessed by the Charlson comorbidity index on the length of stay, costs, and mortality among colorectal cancer patients undergoing colorectal surgery. Curr Med Res Opin. (2023) 39:187–95. doi: 10.1080/03007995.2022.2139053 36269069

[36] Lim J. Big data-driven determinants of length of stay for patients with hip fracture. Int J Environ Res Public Health. (2022) 17:4949. doi: 10.3390/ijerph17144949 PMC7400185 32659953

